# Morse taper performance: A finite element analysis study

**DOI:** 10.6061/clinics/2019/e852

**Published:** 2019-03-19

**Authors:** Gustavo Passarelli Petris, João Paulo De Carli, Luiz Renato Paranhos, Pâmela Letícia Santos, Paula Benetti, Marcio Walber, Eduardo Sandini Linden, Maria Salete Sandini Linden

**Affiliations:** I Departamento de Odontologia, Universidade de Passo Fundo, Passo Fundo, RS, BR; II Departamento de Odontologia Preventiva e Social, Faculdade de Odontologia, Universidade Federal de Uberlandia (UFU), Uberlandia, MG, BR; III Departamento de Ciencias das Saude, Universidade de Araraquara (UNIARA), Araraquara, SP, BR; IV Programa de Pos Graduacao em Engenharia Mecanica, Universidade de Passo Fundo, Passo Fundo, RS, BR

**Keywords:** Dental Implants, Finite Element Analysis, Stress Distribution

## Abstract

**OBJECTIVES:**

To evaluate and compare the magnitude and distribution of stresses generated on implants, abutments and first molar metal-ceramic crowns using finite element analysis.

**METHODS:**

Preliminary three-dimensional models were created using the computer-aided design software SolidWorks. Stress and strain values were observed for two distinct virtual models: model 1 - Morse taper and solid abutment; model 2 - Morse taper and abutment with screw. A load (250 N) was applied to a single point of the occlusal surface at 15° to the implant long axis. Von Mises stresses were recorded for both groups at four main points: 1) abutment-retaining screws; 2) abutment neck; 3) cervical bone area; 4) implant neck.

**RESULTS AND CONCLUSION:**

Model 1 showed a higher stress value (1477.5 MPa) at the abutment-retaining screw area than the stresses found in model 2 (1091.1 MPa for the same area). The cervical bone strain values did not exceed 105 µm for either model.

## INTRODUCTION

Prosthetic connections and their close relationship to peri-implant tissue health are currently the most discussed subject in implantology. Implant longevity is related to the type of retaining screw material, which determines the distribution of stresses originating from masticatory forces. The stresses can cause prosthesis instability, fractures of the retaining screw in single and multiple prostheses and fractures of the implant 1,2.

Reports of prosthesis failure are directly related to material fatigue as a result of cyclic low-intensity loads and slow crack growth. Fatigue 1,2 (the main cause of implant-abutment connection failure) can result in catastrophic fracture of the material when under a load cycle below the stress limit.

Evidence 3 indicates that most fractures in prosthetic structures occur after many years and are related to several episodes of overload, causing failure through fatigue.

Improvements in the design of prosthesis-implant connection decreased but did not eliminate the incidence of mechanical problems 4. Therefore, an adequate number of longitudinal studies is necessary to enhance the safety of these new technologies. Laboratory tests are a reasonable alternative to analyzing material strength and are useful for validating researcher experience. Finite element analysis (FEA), which uses virtual models and environments, is widely used in engineering and computer sciences to simulate and progressively test the strength and stress distribution 5 of machinery components of daily use.

Many health care professionals are applying FEA because it is a high-precision method. FEA consists of dividing an object into finite elements connected by nodes 6. The displacements in any part of the element are expressed as a function of the node displacements. These elements are described by differential equations and solved by mathematical models to obtain the desirable results.

Many studies 2,7,8 have reported the behavior of prosthetic components in response to occlusal efforts, simulating static or cyclic loads applied at an angle or parallel to the long axis of implants.

The high rates of clinical fracture and loss of screws used to retain implant-supported prosthesis is a problem that requires clinicians to have improved knowledge of the biomechanical behavior of implant-supported restorations to properly indicate a solid universal post or a universal post with screw.

Morse taper connections that have been consolidated drastically reduce the number of problems, such as the releasing and loosening of screws, although the intimate contact of the implant/intermediate interface allows voltages to be distributed directly to the adjacent bone, on which the above voltages limit the tolerance that can generate microdanos and bone resorption 8.

Thus, the finite element method is important for analyzing the stress distribution of dental implants and prosthesis elements and contributes to the scientific knowledge of prosthetics. This method can be used to verify the stress concentration areas on the components and correlate these areas to clinical failures. If the high stress concentration areas are identified, the components could be redesigned to overcome weaknesses.

The objective of this study was to qualitatively and quantitatively analyze the behavior of Morse taper connections using a von Mises stress distribution obtained by FEA of three-dimensional (3D) models and to test the hypothesis that solid posts (without trespassing screw) present the lowest stress values.

## MATERIALS AND METHODS

### Model construction

For the analysis, the models were transferred by importing the assembly to the ANSYS Workbench software environment (Swanson Analysis Systems Inc., Houston, PA, USA). This analytical software allows testing of models and parts to predict situations prior to experimental testing to confirm and challenge the experimental test results. Like most analytical software, the ANSYS Workbench provides structural simulation results such as the von Mises equivalent voltage field. The use of ANSYS allows other mechanical properties to be added to the evaluation of the mechanical stimulus. In addition to importing the generated model into SolidWorks in a simple manner, ANSYS generates meshes and elements with the desired mechanical properties. After the forces are applied, these elements are analyzed for the solution of the stimulus.

Two 3D models were built for the application of numerical analysis: model 1 (M1) - Morse taper and solid abutment; model 2 (M2) - Morse taper and abutment with screw (Table 1 shows the description of the analytical models).

The models were created and assembled in SolidWorks version 2011 (SolidWorks Corporation, Santa Monica, CA, USA). SolidWorks is a computer-aided design (CAD) software based on parametric computation and allows the creation of 3D forms from basic geometric shapes. Within the SolidWorks environment, possibilities range from creating a sketch from a digital image obtained by drawing lines or acquiring two-dimensional (2D) or 3D solids from computed tomography (CT) images. In addition, the software allows virtual solids or images to be created and edited, reproducing features of the physical model (original part) with high precision.

The first simplification was the changing of the implant model external grooves to reduce the number of faces and edges, facilitating the subsequent generation of a finite element mesh. The second simplification was the alteration of the implant internal grooving (which retains the abutment), consequently creating two models of the implant because the retaining screws have different dimensions. The third simplification was the subtraction of the abutment chamfers. The fourth simplification was the removal of the coping chamfers. The fifth simplification was the removal of the entries for keys in the abutment. The sixth simplification was the removal of the internal index of the implant because the index presented zero thickness, making the calculation impossible.

To perform the simulation of the assembled model (implant, abutments and prosthesis), bone in the first molar region was modeled using a cross-sectional CT image of the mandible as a template. All analyses were performed using ANSYS Workbench.

### Materials properties

The values of the elastic modulus and Poisson's ratio of the materials were needed to perform the linear static analysis of the models.

The Young's modulus or elastic modulus is a mechanical parameter that provides information on the rigidity of a solid material. When a material is extended, it suffers a longitudinal deformation (determined by the elastic modulus) proportional to the applied stress.

The ratio between the transverse and longitudinal strains in the direction of the tensile force is known as Poisson's ratio.

When stress exceeds a predetermined level, the material undergoes permanent deformation (plastic deformation). The point at which these permanent deformations start to become significant is called the elastic limit (yield point). The values of material mechanical properties 9-11 obtained in this study are presented in Table 2.

### Contact conditions

The areas between cortical and medullary bone, between bone and implant, and between abutments and crowns were considered bonded. The contacting faces between the implant and abutment were considered frictional contact (0.78 friction coefficient^2^).

To define the tensile stress involved, the preload or tensile stress (σt) needed to be calculated. The Falkner model 12 was used in this study (Equation 1). 
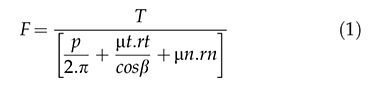


The tensile stress values were 219.3 N for the M1 model and 360 N for the M2 model.

### Mesh discretization

A mesh was generated using 187 solid-type elements, which are 10-node tetrahedral elements that are adequate for irregular forms 5. Table 3 presents the number of nodes and elements for each experimental model.

### Boundary and loading conditions

The supports, defined as fixed (no displacement), were localized in the medial and distal areas of the bone models.

The “bolt pretension” condition was applied to the model. Values of σt or preload obtained earlier were added to the model. Another step was the application of an off-axis loading force of 250 N at 15° to the implant long axis.

To obtain the results, two steps were defined for the analysis:

- 1 s = Preload
- 2 s = Preload + Occlusal force

After the model was set up, solution modes were configured.

The obtained results were reported as von Mises equivalent (EQV) stress values at four main regions: (1) R1 – abutment-retaining screw; (2) R2 – abutment neck; (3) R3 – bone cervical area; and (4) R4 – implant neck.

## RESULTS

The maximum stress magnitudes obtained in the present study (in terms of EQV stress) are shown in Table 4. The distribution of stresses in the abutments and implants at the interfaces for both models (M1 and M2) can be observed in Figures 1A and 1B.

For the implant models, the highest stress levels were observed in the model assembled with a trespassing screw (M2) in the internal neck region (R4) in the direction of the applied force, characteristic of compressive strength. The highest stress values in the region ranged from 500 to 710 MPa, exceeding the yield stress (σ_y_) value of Ti G4 (626 MPa). Small areas presented stress values above the proportionality limit; however, no value exceeded the rupture strength (σ_r_) of Ti G4 (737 MPa).

For the M1 model, stress (ranging from 300 to 500 MPa) was concentrated in the same region. The values found for the M1 model were lower than the σ_y_ of Ti G4 (626 MPa); consequently, no permanent deformation was generated.

**Figure 2:**
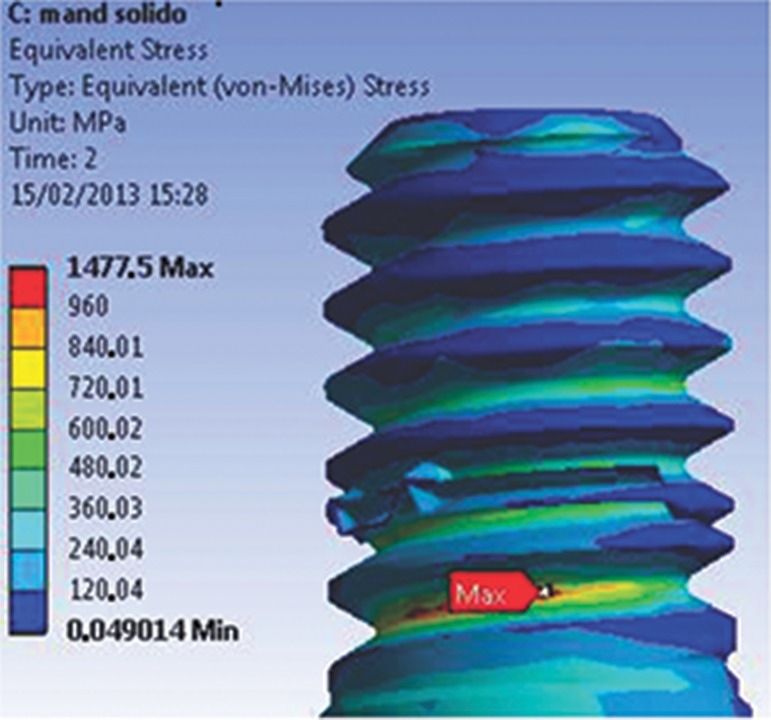
Maximum stress found at R1 (abutment-retaining screw), 2 s, for the M1 model.

The first series of abutment screw grooves (R1) presented the highest stress concentration of the M1 model. The maximum stress value (1475 MPa, far above σ_y_) (Figure 2) corresponds to the region opposite to the direction of the occlusal force application. A possible explanation for the stress values presented by this model to exceed the σ_y_ is the presence of an applied preload or higher σ_t_. Another factor could be related to the model geometry, where specific points can have higher values because of the construction and assembly method.

**Figure 3:**
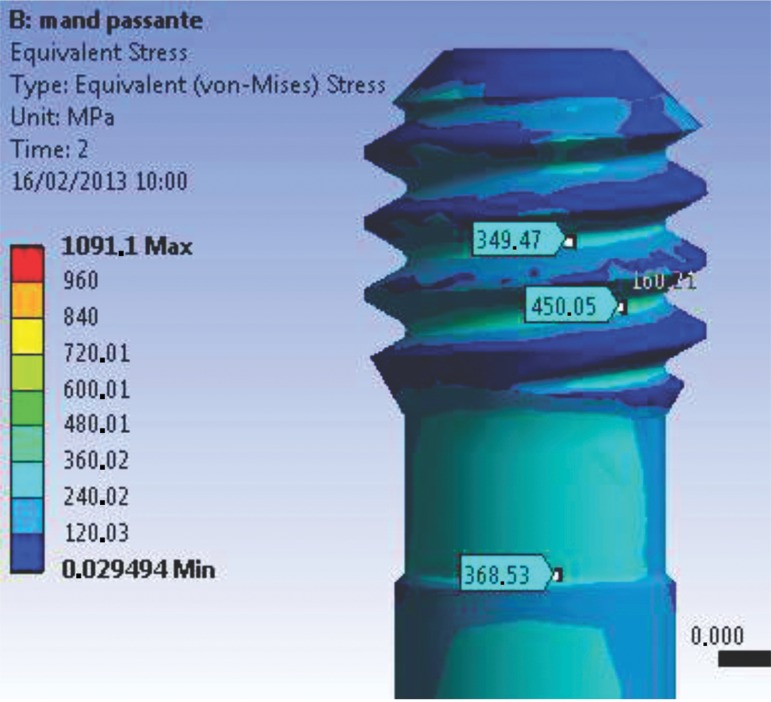
Abutment-retaining grooves (M2), presenting stress concentration values lower than σ_y_.

The abutment-retaining screw in the trespassing screw version of the model had a stable behavior. The geometry provided a uniform flow of stresses through the connection. In addition, the lower σ_t_ for preload application decreased the stress values at the first grooves of the abutment-retaining screw (120 to 450 MPa), as shown in Figure 3. In the upper section of the screw or abutment-retaining screw head, the stress varied from 300 to 555 MPa in the direction of the applied load, combining a safety factor with no negative influence from the preload application.

**Figure 4:**
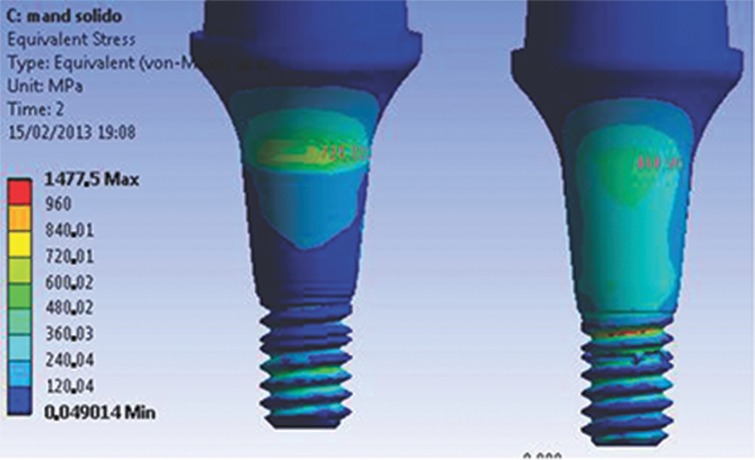
Abutment neck (R2) stress values for the M1 model. Left side – in the direction of the applied force; right side – opposite of the direction of the applied force.

**Figure 5:**
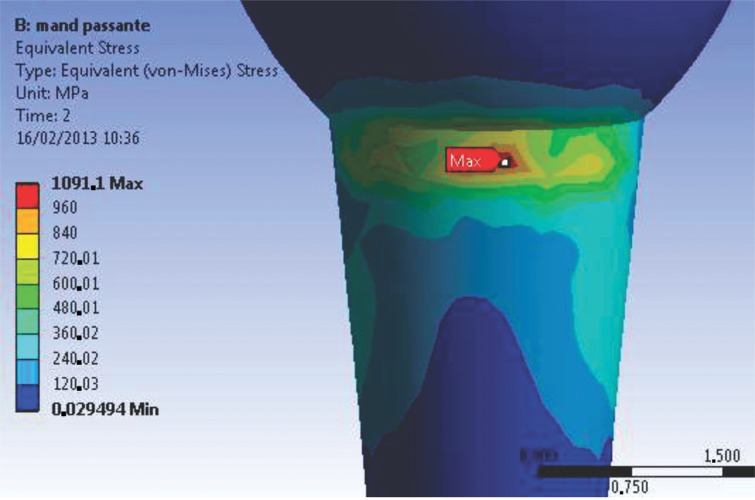
Maximum stress concentration for the M2 model – compressive forces.

The stress concentration values at R2 of the M1 model (1 s time) ranged between 200 and 250 MPa along the extension of the connection. When occlusal loads were applied, these stress values reached 760 MPa in the direction of the applied force (Figure 4), maintaining a safety factor. For the M2 model (Figure 5), the R2 structure walls are thinner and consequently present the maximum stress values of the M2 model. In the direction of the applied load, the stress magnitude was 1091 MPa. However, when the opposite side (tensile force side) was analyzed, the stress values ranged between 155 and 405 MPa, which are below the proportional limit of the material.

No preload application above the σ_y_ of the materials was observed for either model. Figures 6A and 6B show the global stress distribution after preloading.

When the parts of both models were analyzed separately, the beginning of the abutment-retaining screw was observed to be a region of high concentration of stresses during preloading. For both models, the highest stress values, which were lower than the values required for permanent deformation, were concentrated at the deepest portion of the grooves. In addition, the first grooves support most of the stress. At the screw body, the highest stress value was 140 MPa for both models. In this study, the maximum strain values vary from 15 to 105 µm for both the M1 and M2 experimental models (Figures 7A and 7B).

## DISCUSSION

In general, the M1 model showed higher stress values in the first grooves of the abutment-retaining screws. Despite the presence of small areas of deformation, the stress distribution of the implant-abutment-prosthesis-adjacent bone assembly was uniform. The values above the σ_y_, concentrated at the grooves of the retaining screw, are associated with higher preload values.

The M2 model presented the lowest stress values, showing mechanical behavior with lower values above the proportionality limit. However, the tapered neck region presented a stress concentration above the rupture limit with large areas of permanent deformation. The applied preload is sufficient to maintain the abutment in position, and friction is responsible for the connection security, allowing free displacement conditions through the retaining screw and the implant neck.

The clinical implications of the models showed that both solid and trespassing posts had strain values varying from 15 to 105 µm, characteristic of a beneficial behavior (stimulation) for bone tissue. The solid post is more effective at dissipating stress and is safer for clinical use.

The biomechanical performance of the implant-abutment-prosthesis-adjacent bone assembly in the functional environment is very different and depends on the prosthetic connection chosen by the dentist. The Morse taper connections reduce mechanical problems 13, such as the loosening and loss of the screw, because of a frictional resistance; thus, these connections are safer.

The slope of the inner cone promotes a more uniform dissipation of stress at the implant-abutment-prosthesis-adjacent bone assembly 13. This connection is safer because of the high pressure between the homologous conical walls of the abutment and implant and the resulting frictional resistance 2.

In addition to mechanical safety, the biological safety of this connection is of great importance. The presence of any vertical connection mismatch and thin prosthetic structure walls can generate high stress concentration areas 14.

The universal abutment model with trespassing screw showed similar behavior as the model with thin component structure walls, which promoted high stress concentrations with stress values above the σ_y_, as described in a previously mentioned study.

Based on the Falkner method 12, the material friction coefficient is inversely proportional to the generated preload. This relationship shows the importance of applying the correct torque to the trespassing screw and of the need to follow the manufacturer's instructions.

The most reliable analysis of bone behavior is based on the values of EQV strain, representing the displacement behavior of the model. A displacement greater than 150 µm is harmful for the bone: fibrous encapsulation can occur or osseointegration can be lost 15,16. Conversely, a displacement between 15 and 105 µm has a positive influence on osseointegration 17, stimulating the deposition of bone with better mechanical qualities. The highest strain values for both models did not exceed 105 µm, demonstrating that both regions have a similar behavior independent of the type of retaining screw in the abutment-Morse connection, even though the solid universal post can smoothly distribute the stress generated by the occlusal force through the connection and the marginal bone.

The behavior obtained in the M1 model, the solid version of the universal post, showed that the connection is safe and effective for dissipating stress, and the behavior of the two models was very similar when the load was applied at the opposite side. The M2 model with trespassing screw showed higher values of EQV stress in the neck of the post.

The behavior of the connection obtained in the solid trunnion model was shown to be safer and more efficient when incorporating the flow of the resulting applied occlusal force. The two models of universal trunnion had values and behavior towards the opposite side to the application of the load very similar; however, in the model with the bolt, the trunnion neck presented values above the limit of rupture on the side of compression (direction of loading).

Future studies related to the finite element method should guarantee a more precise construction phase of the models, considering the different types of behavior of the materials of anisotropic characteristics, in addition to proposing dynamic and nonlinear analyses because the behavior of human tissues can be simulated, thus bringing the results compared to the different clinical situations.

## AUTHOR CONTRIBUTIONS

Petris GP and Linden ES were responsible for the acquisition of data, analysis and interpretation of data, manuscript writing and final approval. Linden MS and De Carli JP were responsible for the conception and design of the study and manuscript final approval. Paranhos LR was responsible for the definition of intellectual content, critical revision and manuscript final approval. Santos PL was responsible for the manuscript preparation and editing, critical revision and manuscript final approval. Benetti P was responsible for the acquisition of data, analysis and interpretation of data, manuscript writing and final approval. Walber M was responsible for the analysis and interpretation of data, manuscript critical revision and final approval.

## Figures and Tables

**Figure 1A:**
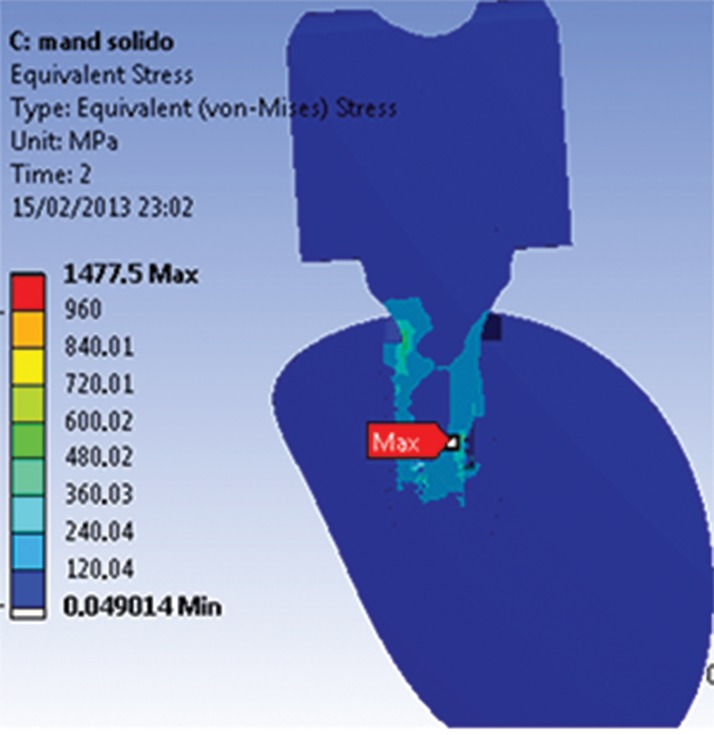
Von Mises stress results showing the point of maximum stress for M1.

**Figure 1B:**
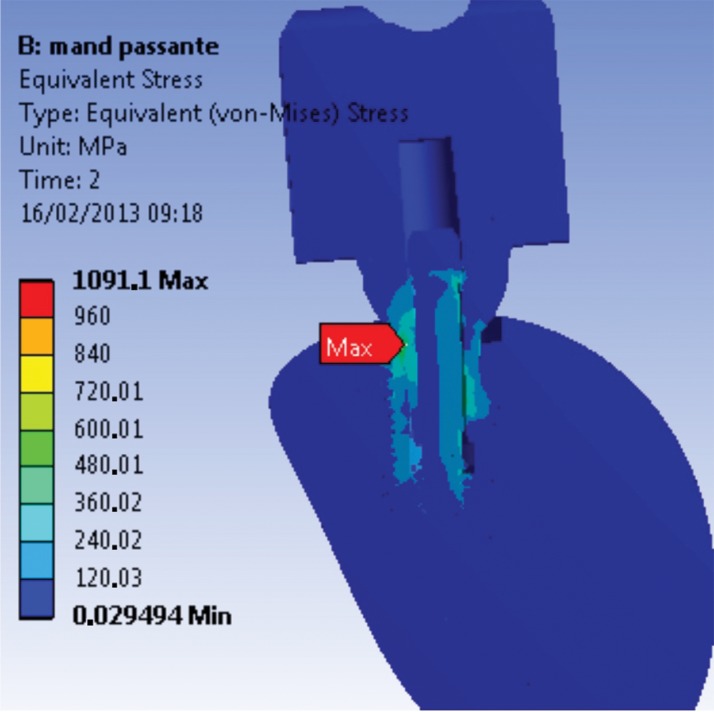
Von Mises stress results showing the point of maximum stress for M2.

**Figure 6A:**
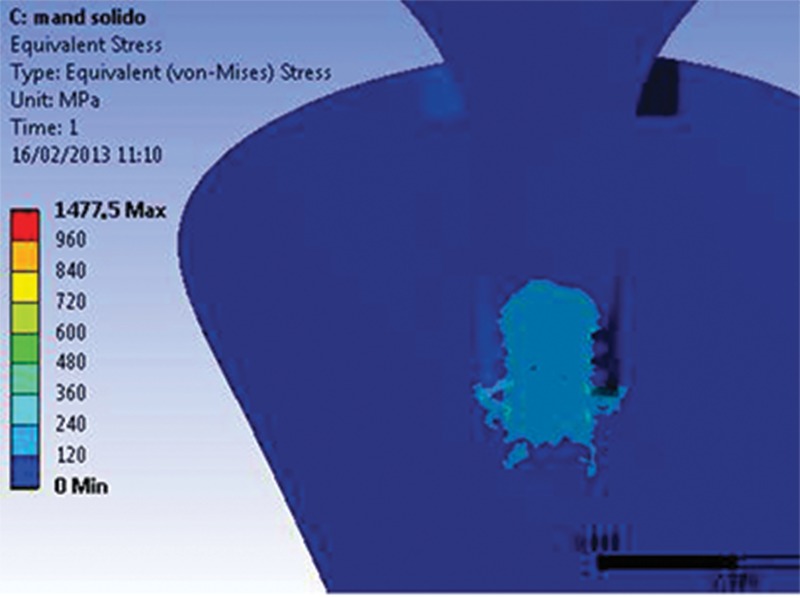
Preloading effect on the M1 model.

**Figure 6B:**
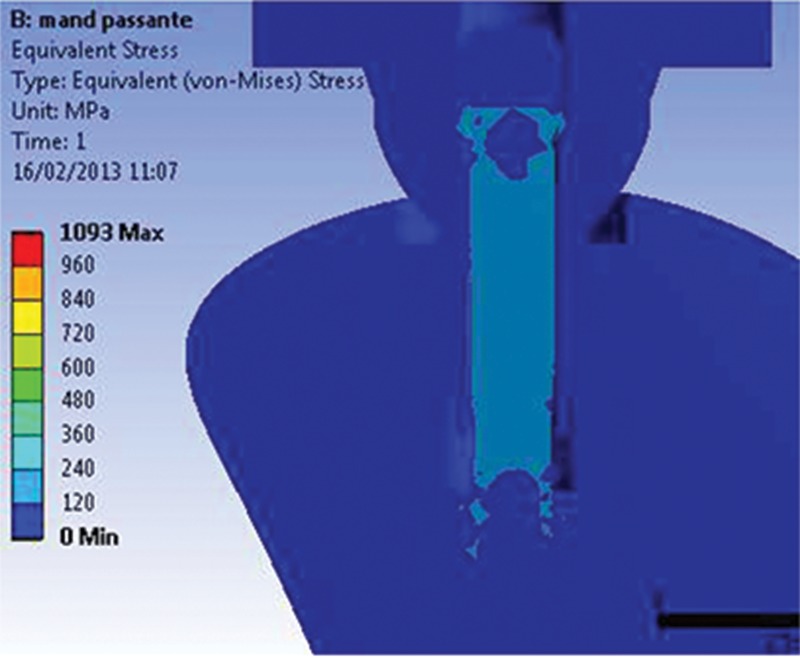
Preloading effect on the M2 model.

**Figure 7A:**
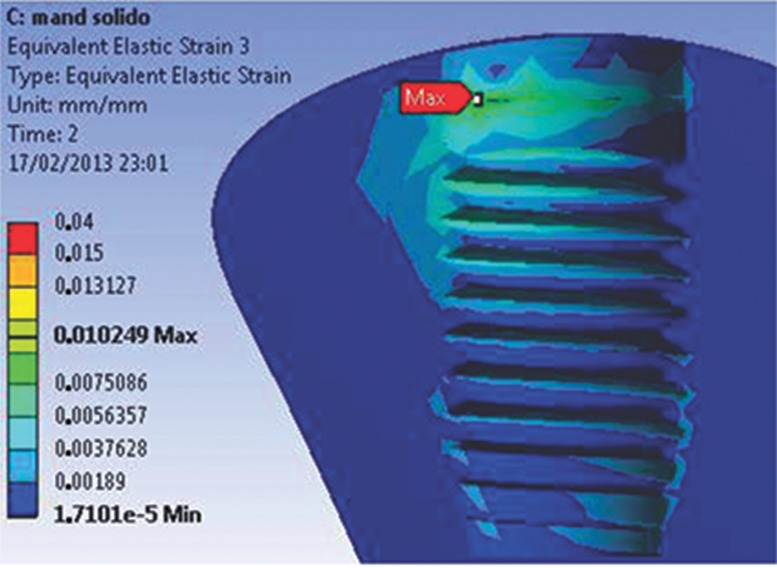
EQV strain for the M1 model.

**Figure 7B:**
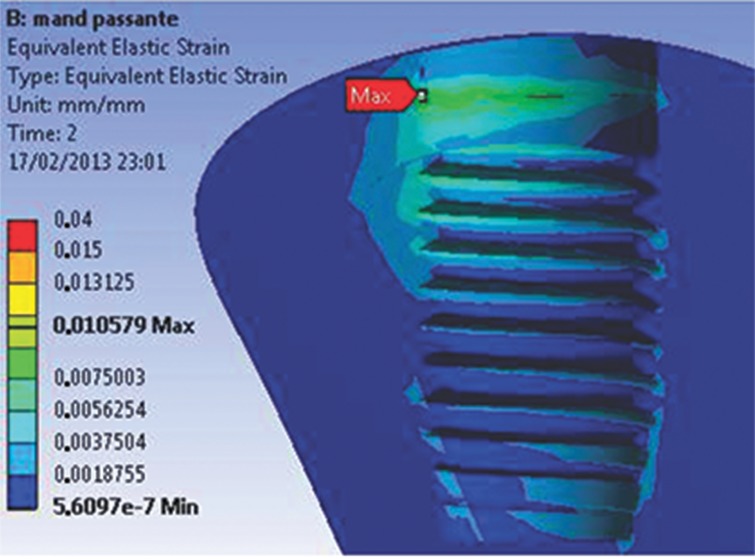
EQV strain for the M2 model. Notably, the strain is higher for this model; however, the average values are similar to those of the M1 model.

**Table 1:** Description of the study models.

	Bone Area	Implant Dimensions	Connection	Abutment	Prostheses
**M1**	Mandible 1^st^ Molar	3.75 x 7 mm	Morse taper 11.5°	Solid Universal Post	Metal-ceramic
**M2**	Mandible 1^st^ Molar	3.75 x 7 mm	Morse taper 11.5°	Universal post with screw	Metal-ceramic

**Table 2:** Material mechanical properties.

	Elastic modulus (ε) MPa	Poisson’s ratio (υ)	Yield point (σ_y_) MPa
**Cortical bone**	14.000	0.30	60-70
**Medullary bone**	1.000	0.30	-
**Ti G4**	105.000	0.34	626
**Ti6Al4V**	110.000	0.34	960
**Cr - Co**	218.000	0.33	900
**Feldspatic ceramic**	68.900	0.28	69

**Table 3:** Number of nodes and elements for each experimental model.

	M1		M2	
	Nodes	Elements	Nodes	Elements
**Total**	123218	72983	117197	68440

**Table 4:** Maximum stress values for each experimental model.

Model	M1				M2			
	R1	R2	R3	R4	R1	R2	R3	R4
Preload (MPa)	460.51	204.4	X	140	574.55	X	X	150
Preload + Occlusal force (MPa)	1477.55	760	0-80	512	450	1091	0-80	1000

## References

[1] Wiskott HW, Nichols JI, Belser UC (1995). Stress fatigue: basic principles and prosthodontic implications. Int J Prosthodont.

[2] Geramy A, Morgano SM (2004). Finite element analisys of three designs of an implant-supported molar crown. J Prosthet Dent.

[3] Binon PP (2000). Implants and components: entering the new millennium. Int J Oral Maxillofac Implants.

[4] Coppedê AR, Bersani E, De Mattos Mda G, Rodrigues RC, Sartori IA, Ribeiro RF (2009). Fracture resistance of the implant-abutment connection in implants with internal hex and internal conical connections under oblique compressive loading: an in vitro study. Int J Prosthodont.

[5] Alves A (2000). Elementos finitos: A base da tecnologia CAE.

[6] Geng JP, Tan KB, Liu GR (2001). Application of finite element analysis in implant dentistry: A review of the literature. J Prosthet Dent.

[7] Merz BR, Hunenbart S, Belser UC (2000). Mechanics of the implant-abutment connection: an 8-degree taper compared to a butt joint connection. Int J Oral Maxillofac Implants.

[8] Pessoa RS, Muraru L, Júnior EM, Vaz LG, Sloten JV, Duyck J (2010). Influence of implant connection type on the biomechanical environment of immediately placed implants - CT-based nonlinear, three-dimensional finite element analysis. Clin Implant Dent Relat Res.

[9] Anusavice KJ (1996). Phillips’ Science of Dental Materials. 10th Edition.

[10] Kelly JR, Nishimura I, Campbell SD (1996). Ceramics in dentistry: Historical roots and current perspectives. J Prosthet Dent.

[11] Moaveni S (2003). Finite element analysis: theory and application with ANSYS.

[12] Bickford JH (1990). An Introduction to the Design and Behavior of Bolted Joints.

[13] Bozkaya D, Muftu S (2003). Mechanics of the tapered interference fit in dental implants. J Biomech.

[14] Bacchi A, Consani RL, Mesquita MF, Dos Santos MB (2013). Effect of framework material and vertical misfit on stress distribution in implant-supported partial prosthesis under load application: 3-D finite element analysis. Acta Odontol Scand.

[15] Brunski JB (1992). Biomechanical factors affecting the bone-dental implant interface. Clin Mater.

[16] Geris L, Andreykiv A, Van Oosterwyck H, Sloten JV, van Keulen F, Duyck J (2004). Numerical simulation of tissue differentiation around loaded titanium implants in a bone chamber. J Biomech.

[17] Vandamme K, Naert I, Geris L, Vander Sloten J, Puers R, Duyck J (2007). The effect of micro-motion on the tissue response around immediately loaded roughened titanium implants in the rabbit. Eur J Oral Sci.

